# Mapping Iterative Medical Imaging Algorithm on Cell Accelerator

**DOI:** 10.1155/2011/843924

**Published:** 2011-09-11

**Authors:** Meilian Xu, Parimala Thulasiraman

**Affiliations:** Department of Computer Science, University of Manitoba, Winnipeg, MB, Canada R3T 2N2

## Abstract

Algebraic reconstruction techniques require about half the number of projections as that of Fourier backprojection methods, which makes these methods safer in terms of required radiation dose. Algebraic reconstruction technique (ART) and its variant OS-SART (ordered subset simultaneous ART) are techniques that provide faster convergence with comparatively good image quality. However, the prohibitively long processing time of these techniques prevents their adoption in commercial CT machines. Parallel computing is one solution to this problem. With the advent of heterogeneous multicore architectures that exploit data parallel applications, medical imaging algorithms such as OS-SART can be studied to produce increased performance. In this paper, we map OS-SART on cell broadband engine (Cell BE). We effectively use the architectural features of Cell BE to provide an efficient mapping. The Cell BE consists of one powerPC processor element (PPE) and eight SIMD coprocessors known as synergetic processor elements (SPEs). The limited memory storage on each of the SPEs makes the mapping challenging. Therefore, we present optimization techniques to efficiently map the algorithm on the Cell BE for improved performance over CPU version. We compare the performance of our proposed algorithm on Cell BE to that of Sun Fire ×4600, a shared memory machine. The Cell BE is five times faster than AMD Opteron dual-core processor. The speedup of the algorithm on Cell BE increases with the increase in the number of SPEs. We also experiment with various parameters, such as number of subsets, number of processing elements, and number of DMA transfers between main memory and local memory, that impact the performance of the algorithm.

## 1. Introduction

Medical imaging such as X-ray computed tomography has revolutionized medicine in the past few decades. The use of X-ray computed tomography has increased rapidly since 1970 when Radon's technique for reconstructing images from a set of projections was first introduced in the medical field. In 2007, it was estimated that more than 62 million scans per year were obtained in United States and about 4 million for children [1]. The number of scanners has also increased in many countries due to the ease of using these machines. The commonly used analytic technique in CT scanners to produce diagnostic evaluation of an organ or the region of interest is Fourier back projection (FBP). This technique requires a large number of projections measured uniformly over 180° to 360° [2], inducing a large amount of radiation into the body to produce quality images. Therefore, there has been a lot of interest in developing algorithms that minimize the radiation dose without impairing image quality. One such class of algorithms [2, 3] that has been studied are iterative or algebraic algorithms.

Theoretically, iterative methods require about half of the number of projections as that of FBP method [4], which makes these methods safer in terms of required radiation dose. Compared to FBP method, iterative methods have the added advantage of producing reconstructions of better quality when data is incomplete, dynamic, or noisy. These methods solve a linear system of equations and pass through many iterations of computation to reconstruct the image. Each equation corresponds to a ray. A projection angle comprises many such rays within the same angle. For the purpose of illustration, we assume *Q* projection angles and *M* rays in total.

There are basically four steps in the iterative reconstruction algorithm: (i) forward projection, (ii) error correction, (iii) back projection, and (iv) image update. The algorithm terminates when the convergence criterion is satisfied. There are several iterative algorithms in the literature. These algorithms all follow the four steps mentioned above but differ as to when the image updates are performed. The number of updates determines the quality of the image and also gives an upper bound on the total computation time [5]. We believe that we can make use of the tremendous amount of raw computational power available on Cell BE to provide faster performance and convergence of iterative algorithms. We assume in this paper that an iteration comprises steps (i) to (iii) followed by an image update.

Historically, the algebraic reconstruction technique (ART), a ray-based method, proposed by Gordon et al. [6], is the first representative iterative algorithm. ART iterates through the three steps (one iteration) for each ray, and then updates the image at the end of step three. Note that an image update is done for each ray which is highly time consuming. Also, this is very sequential in nature. Simultaneous iterative reconstruction technique (SIRT) [7] improves upon ART and iterates through steps (i) to (iii) for all the rays before performing an image update. This method requires many iterations for accurate results and, therefore, has a slower convergence rate. Simultaneous algebraic reconstruction technique (SART) [8] combines the good properties of ART and SIRT. The algorithm works on projections. SART passes through steps (i) to (iii) for rays within one projection, followed by an image update. This is done iteratively for each of the *Q* projections. Note that since the image is updated after computing the rays of each of the *Q* projections, the convergence rate is faster and the number of iterations compared to SIRT is reduced. Both SART and SIRT produce better-quality images than ART. However, they are computationally intensive. The convergence rate of simultaneous methods can be further accelerated through ordered-subsets (OS) technique [1, 9]. Ordered subsets method partitions the projection data into disjoint subsets and processes the subsets sequentially. For ART, each ray corresponds to one subset. Therefore, for *M* rays, there are *M* subsets. In the case of SIRT, all rays (*M*) correspond to one subset only. A subset in SART may correspond to all the rays in one projection angle or combine several projections of different angles into one subset. This is called OS-SART [10]. Due to the fast convergence rate of SART, in this paper, we consider parallelization of SART using the ordered-subsets (OS) technique. Though OS-SART can reduce the reconstruction time with respect to the convergence rate and produce images with high quality, it is still prohibitively time consuming due to its computation-intensive nature, especially for large images with high-resolution requirements.

One approach to increase the performance of the OS-SART algorithm is to parallelize the algorithm on modern heterogeneous multicore systems which aim to reduce the gap between the application required performance and the delivered performance [11]. The cell broadband engine (Cell BE) [12] is one such architecture. This architecture supports coarse-grained data parallel applications. In OS-SART each of the subset performs the same algorithm (same instructions) supporting data parallelism.

In this paper, we use a domain-decomposition method, which subdivides the problem into disjoint subsets. In OS-SART, each subset has to be computed iteratively. Therefore, the projection angles are further subdivided and assigned to the synergetic processor elements (SPE). Each SPE can compute independently and concurrently without any communication, reducing communication overhead. Due to the limited local store on each of the SPEs, we incorporate optimization techniques in OS-SART.

The paper is organized as follows.  Section 2 lists a selection of related work.  Section 3 gives a brief introduction to iterative reconstruction techniques and OS-SART algorithm.  Section 4 analyzes the properties and complexities of OS-SART and introduces the rotation-based projector and back-projector used in OS-SART algorithm for this paper.  Section 5 lists the highlights of Cell processor, with the Cell-based OS-SART algorithm followed in Section 6. Experiment results are given in Section 7, followed by discussions in Section 8.  Section 9 will conclude this paper.

## 2. Related Work

Compared to parallel computing research on analytic techniques, research on iterative techniques is few. Laurent et al. [13] parallelized the block-ART and SIRT methods for 3D cone beam tomography. The authors developed a fine-grained parallel implementation, which introduced more frequent communications and degraded the performance of their algorithm. Backfrieder et al. [14] used web-based technique to parallelize maximum-likelihood expectation-maximization (ML-EM) iterative reconstruction on symmetric multiprocessor clusters. A java-applet enabled web-interface was used to submit projection data and reconstruction tasks to the cluster. Li et al. [15] parallelized four representative iterative algorithms: EM, SART and their ordered subsets (OS) versions for cone beam geometry on a Linux PC cluster. They used micro-forward-back-projection technique to improve the parallelization at the ray level during forward projection. 

Gordon [16] parallelized 2D ART using a linear processor array. The author investigated both sequential ART and parallel ART algorithm on different phantom data with or without noise introduced for different number of projection views. Kole and Beekman [17] parallelized ordered subset convex algorithm on a shared memory platform and achieved almost linear speedup. Melvin et al. [18] parallelized ART on a shared memory machine and observed that the speedup of ART on shared memory architectures was limited due to the frequent image update of the ray-based ART algorithm. The parallel algorithm incurred communication and synchronization overheads and degraded the performance. Subsequent to this work, Xu and Thulasiraman [19] considered the parallelization of OS-SART on shared memory homogeneous multicore architecture. The OS-SART algorithm produces higher granularity for parallelization to reduce the above-mentioned communication and synchronization latencies. The algorithm was experimented on a CPU-based shared memory machine which provides only few dozen nodes. Due to synchronization and communication overheads of shared memory machines, the authors were unable to produce improved performance gain. In this work, the algorithm takes advantage of Cell BE's architecture: the SPEs (coprocessors) compute fine grained independent tasks, while the PPE performs the tedious tasks of gathering and distributing data. We overlap computation and communication through mechanisms such as direct memory access available on the Cell BE to tolerate synchronization and communication overheads.

Mueller and Yagel investigated SART [20], SIRT, and OS-SIRT on an older heterogeneous multicore GPU hardware. They found that the special architecture and programming model of GPU adds extra constraints on the real-time performance of ordered subset algorithms. Xu et al. [21] recently implemented OS-SIRT and SART on the GPU architecture and claimed that SART or its subsequent OS-SART is not a suitable algorithm for implementation on GPU and does not provide increased performance gain though the convergence rate is faster than SIRT.

In this paper, we show that OS-SART algorithm is suitable for parallelization on the Cell BE and compare the results to our earlier work on homogeneous multicore architecture [19].

## 3. Iterative Reconstruction Techniques and OS-SART Algorithm

**Figure 1:**
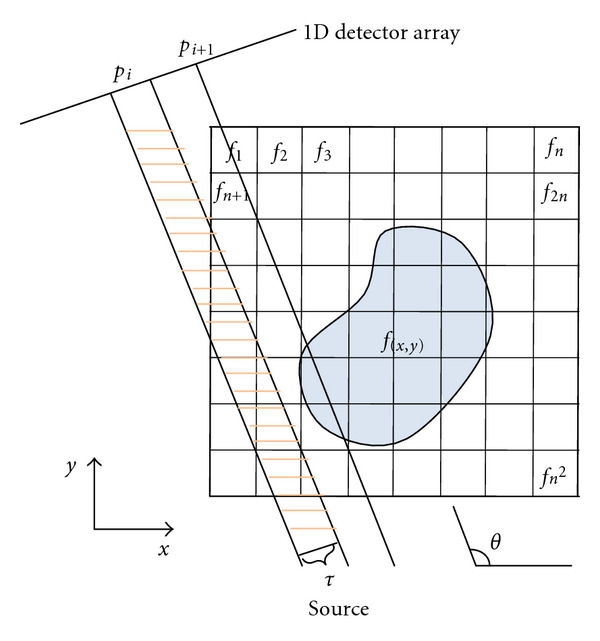
Illustration of iterative methods.

In this section, we start with an illustration of the iterative reconstruction technique. In Figure 1, *f*(*x*, *y*) is an unknown image of an object and *p*_*i*_ is a ray of one projection at an angle *θ*. Many such projection data may be acquired via scanners. In this paper, we assume that 1D detector array is used to acquire projection data by impinging parallel beams onto a 2D object. The object is superimposed on a square grid of *N* = *n*^2^ cells, assuming each cell is made up of homogeneous material having a constant attenuation coefficient value *f*_*j*_ in the *j*th cell [2]. A ray is a strip of width *τ* in *x*-*y* plane as shown in Figure 1. In most cases, the ray width *τ* is approximately equal to the cell width. A line integral along a particular strip is called *raysum*, which corresponds to the measured projection data in the direction of that ray. A projection (or view or projection view) is defined as all rays projecting to the object at the same angle.

Let *p*_*i*_ be the *raysum* measured for ray *i* as shown in Figure 1. Assume that all raysums are represented using one-dimensional array. The reconstruction problem can be formulated to solve a system of linear equations as follows:

(1) $$\begin{array}{c} \overset{N}{\underset{j=1}{\sum }}w_{ij}f_{j}=p_{i},\;i=1,2,\ldots ,M, \end{array}$$

where *M* is the total number of rays. *w*_*ij*_ is the weighting factor that represents the contribution of *j*th cell along the *i*th ray. The weighting factor can be calculated as (i) the fractional area of the *j*th cell intercepted by the *i*th ray or (ii) the intersection length of the *i*th ray by *j*th cell when the ray width *τ* is small enough to be considered as a single line. In this paper, we use the latter (Siddon's method) and will be explained in the next section. Note that for different rays, *w*_*ij*_'s have different values for the same *j*th image cell. The left side of each equation in (1) is used as the forward projection operator for the specific ray *i*. In Figure 1, most of *w*_*ij*_'s are zero since only a small number of cells contribute to any given *raysum*. For example, there are only ten nonzero *w*_*ij*_'s for projection *p*_*i*_ if we consider using the fractional areas as the contributions.

**Figure 2:**
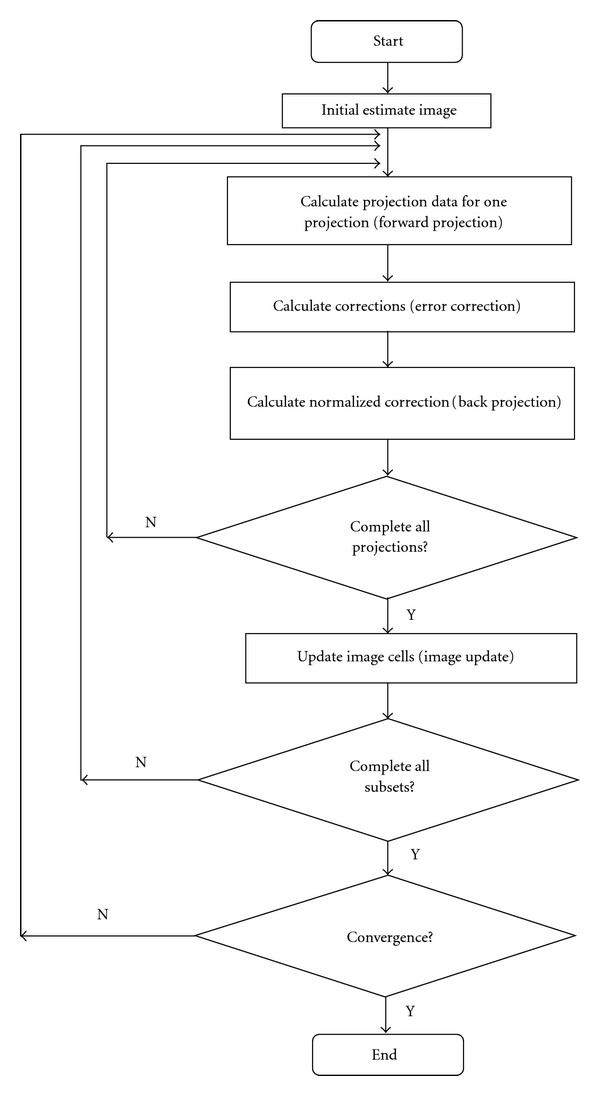
Framework of OS-SART reconstruction technique.

All the rays in one projection corresponds to one subset in SART. In OS-SART, a subset may consist of many such projections.  Figure 2 shows a flow chart for OS-SART. The algorithm iterates over many ordered subsets sequentially before checking the convergence criterion. The image cells are updated with

(2) $$\begin{array}{c} f_{j}^{r,l+1}=f_{j}^{r,l}+\lambda \cdot \frac{\sum_{i\in \text{O}{\text{S}}_{\text{l}}}\left[\left(p_{i}-\sum_{k=1}^{N}w_{ik}f_{k}^{r,l}\right)/\sum_{k=1}^{N}w_{ik}\right]\cdot w_{ij}}{\sum_{i\in \text{O}{\text{S}}_{\text{l}}}w_{ij}},\\ \;\;\;\;\;\;\;\;\;\;\;\;\;\;\;\;\;\;j=1,2,\ldots N, \end{array}$$

where *p*_*i*_ is the raysum of ray *i*, *w*_*ij*_ is the weighting factor, *r* is the iteration index, and *l* is the subset index. *λ* is a relaxation parameter used to reduce noise. Let, corresponding subset index (CIS), CIS = {1,2,…, *Q*} correspond to indices of *Q* projections for the total of *M* rays. CIS is partitioned into *T* nonempty disjoint subsets *OS*_*l*_, 0 ≤ *l* < *T*.

Recall that each subset is computed iteratively. The computation of the pixel values for a subset, *l* + 1 requires that the subset *l* has already been computed and the image has been updated. Using this updated image, (2) is computed. As you can see, *f*_*j*_^*r*,*l*+1^ depends on the weighting factors *w*_*ij*_ and the pixel values computed for the subset *l*, *f*_*j*_^*r*,*l*^. Therefore, although there is synchronization between subsets, there is no synchronization within a subset. We exploit this parallelism on Cell BE.

The image estimate for each angle can be stored in main memory. The correction ((*p*_*i*_ − ∑_*k*=1_^*N*^*w*_*ik*_*f*_*k*_^*r*,*l*^)/∑_*k*=1_^*N*^*w*_*ik*_) and back projection (∑_*i*∈OS_l__[(*p*_*i*_ − ∑_*k*=1_^*N*^*w*_*ik*_*f*_*k*_^*r*,*l*^)/∑_*k*=1_^*N*^*w*_*ik*_] · *w*_*ij*_/∑_*i*∈OS_l__*w*_*ij*_) for the subset is a cumulative result of correction and back projection of different angles in the subset of the current iteration. Therefore, these can be done in parallel also. The only step that requires sequential computation in (2) is the image update. This step requires the cumulative result of the correction and back projection contributions from all angles in the subset.

The calculation of weighting factors are not only computationally intensive, they are also memory bound. In the next section, we use a technique that saves on memory and computation for efficient computation on Cell BE.

## 4. Optimization Techniques on Cell BE

The sequential OS-SART algorithm is presented in Figure 2. The forward projection and back projection steps are the most time-consuming parts of the algorithm. The computation complexity of each step is: *O*(*I* × *T* × *Q*/*T* × *n*^2^) = *O*(*I* × *Q* × *n*^2^), where *I* is the total number of iterations. Let *Q* = *n*. Then, the computation complexity of the algorithm is *O*(*n*^3^), making OS-SART computationally intensive. The OS-SART algorithm is also memory bound. The memory requirement for the forward projection step includes the space required for storing the weighting factors matrix (*w*_*ij*_) for one subset and the entire image. The space for the matrix and the image are *O*(*M*/*T* × *n*^2^) and *O*(*n*^2^), respectively. Since *M* normally has the same magnitude as *N* = *n*^2^ [2], the memory complexity of OS-SART is *O*(*n*^4^), making this algorithm memory intensive.

Typically, the detector array is rotated around an image, and the matrix is computed for all rays within a projection angle. For *Q* projections, there will exist *Q* such matrices. In general, the matrix *w*_*ij*_ is quite large. On the Cell BE, we are limited by the amount of memory available on each of the SPEs. Although, we could store the values in main memory, transferring data from main memory to local stores in SPE a few chunks at a time, it will degrade the performance of the algorithm due to intensive communication overhead. Therefore, in this paper, we use a rotation-based algorithm [22, 23] that is less sensitive to memory. In this method, the image is rotated around the detector array (instead of the detector array being rotated around the image) at a base angle *θ*. The values of *w*_*ij*_ are calculated for this angle and stored as reference. Let us call this *w*_*ij*_^base^. This is a one-time computation. To calculate the projection values at an angle *θ*_*i*_, the forward projection starts by rotating the object at angle *θ*_*i*_ using bilinear interpolation method. The method then computes the forward projection data by summing over all nonzero pixels along each ray in the rotated image. That is, the pixel values are calculated using the reference matrix, *w*_*ij*_^base^ and the rotated image. The back projection starts with the traditional back projection process, followed by rotating the object back with −*θ*_*i*_. Note that the main memory only stores one base weighting factor matrix which is significantly less than storing *Q* weighting factor matrices as in nonrotation-based methods.

As mentioned in the previous section, there are two ways of calculating the weighting factors. In this paper, we use Siddon's method [24], since it reduces the computing complexity from *O*(*N*^3^) of the general ray tracing method to *O*(3*N*).

## 5. Cell Broadband Engine

The Cell BE processor is a chip multiprocessor (CMP) with nine processor elements, one PPE and eight SPEs, operating on a modified shared memory model [25]. Other important architectural features include a memory controller, an I/O controller, and an on-chip coherent bus EIB (element interconnect bus) which connects all elements on the single chip. The SPU (synergistic processing unit) in an SPE is a RISC-style processing unit with an instruction set and a microarchitecture. The PPE is the typical CPU, 64-bit PowerPC architecture which provides operating system support. The eight SPEs are purposely designed for high performance data-streaming and data-intensive computation via large number of wide uniform registers (128-entry 128-bit registers). One of the drawback of the Cell BE is the small amount of private local store (256 KB) available on each of the SPEs.

The most important difference between the PPE and SPEs is the way they access the main memory. PPE accesses the main memory directly with load and store instructions that move data between the main memory and a private register file, just like conventional processors access main memory. On the other hand, SPEs cannot access the main memory directly. They have to issue direct memory access (DMA) commands to move data and instructions between the main memory and their own local store. However, DMA transfers can be done without interrupting the SIMD operations on SPEs if the operands of SIMD operations are available in the local store. This 3-level organization of storage (register file, local store, and main memory), with asynchronous DMA transfers between local store and main memory, is a radical difference from conventional architectures and programming models [25] which complicates the programming effort by requiring explicit orchestration of data movements.

## 6. Cell-Based OS-SART Algorithm

**Figure 3:**
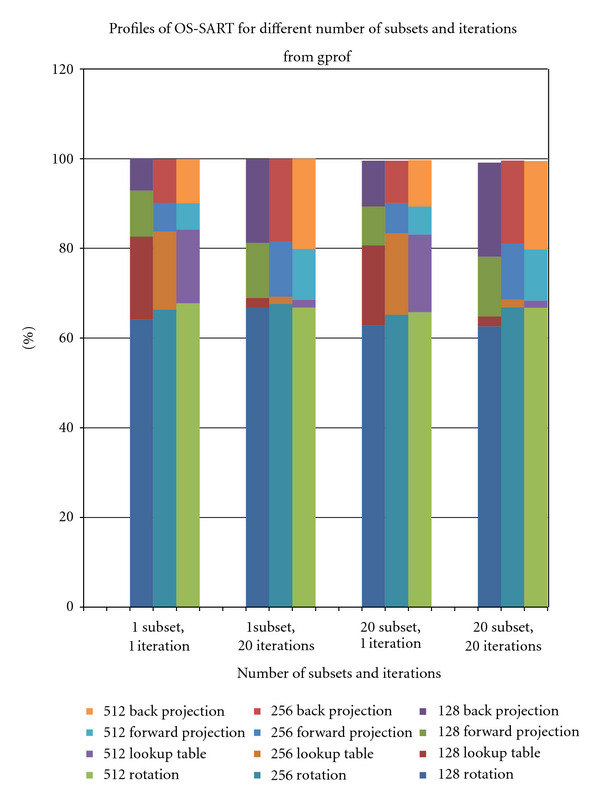
Profile results of OS-SART.

There are four important routines in our proposed rotation-based OS-SART algorithm: forward projection, rotating the image, back projection, and creating reference matrix *w*_*ij*_^base^. By using a profiling tool, gprof, we determined the percentage of execution time spent on these routines. This was done to determine which routines require more effort in parallelization.  Figure 3 shows the results for these routines for varying image sizes, with 20 subsets for 1 and 20 iterations. For both iterations, we notice that the rotation of the image is the most time consuming part. For 20 iterations, the forward projection, back projection, and rotation are also time consuming. The creation of the reference matrix is negligible. Therefore, from this figure we can see that forward projection, back projection, and rotation require efficient parallelization.

On the Cell BE, the creation of the reference matrix is computed by the PPE and stored in main memory. This is a one-time computation. The PPE controls the algorithm. It also assigns the projection angles to each of the SPEs. Given *Q* projection angles and *T* subsets, *Q*/*T* projection angles are assigned to each subset. The angles within the subset, *OS*_*l*_, are further divided. For *P* SPEs, each SPE is assigned *Q*/(*T*∗*P*) projection angles. This process is repeated for each subset. The PPE schedules the angles to the SPEs. At the end of the calculation of SPEs on a subset, the PPE performs the image update and assigns angles from the next subset, *OS*_*l*+1_, to each SPE.

Each of the SPEs performs the following computations for their assigned angles *θ*_*j*_. First, it rotates the image at an angle *θ*_*j*_. Then, it computes the forward projection by accessing the reference weighting factor matrix and the image from main memory via asynchronous DMA transfers. Due to the limited local store in each of the SPEs, the matrix and image are accessed in chunks. Transferring data from main memory to local store is called DMAin [25]. Depending on the size of the image, this process may take several rounds. In the next step, the SPEs perform the error correction at the end of the forward projection computation. After error correction step, the SPE performs the back projection. The SPE sends the data back to main memory in chunks, called DMAout [25]. This is again due to the limited memory on each SPE. Finally, the SPE rotates the image back to its original position and stores this in main memory. The above process is done by an SPE for each of its assigned angles. In our paper, we balance the load on each of the SPEs by assigning the same number of projection angles.

Algorithms 1 and 2 show the pseudocode of the PPE and SPE algorithms discussed above.

## 7. Evaluation and Results

We have tested our proposed algorithm on two architectures: Cell BE and Sun Fire x4600. The Cell BE [26] is PowerXCell 8i processor in IBM QS22 Blade. It runs at 3.2 GHz with 16 GB of shared memory. The compiler is IBM xlc for both PPU and SPU. The Sun Fire x4600 is a distributed shared memory machine with eight AMD dual-core Opteron processors (16 cores in total) running at 1 GHz with 1 M cache per core and 4 GB memory per processor. OpenMP [27] is used for this environment.

The projection data is obtained from CTSim simulator 3.0.3 [28]. CTSim simulates the process of transmitting X-rays through phantom objects. In this work, we focus on 2D images to test the feasibility of our proposed parallel OS-SART algorithm. We use the Shepp-Logan phantom image of size 256 × 256, and 360 projections over 360 degrees. The choice of a relaxation factor has an impact on the number of iterations and reconstruction quality. The relaxation factor is usually chosen within the interval (0.0,1.0] [5]. Mueller et al. note that a smaller *λ* provides a less noisy reconstruction but may increase the number of iterations for convergence. The authors through their experiments conclude that *λ* within the interval [0.02,0.5] produces better reconstruction images with less number of iterations. Therefore, in this paper, we experiment with *λ* = 0.2.

**Figure 4:**
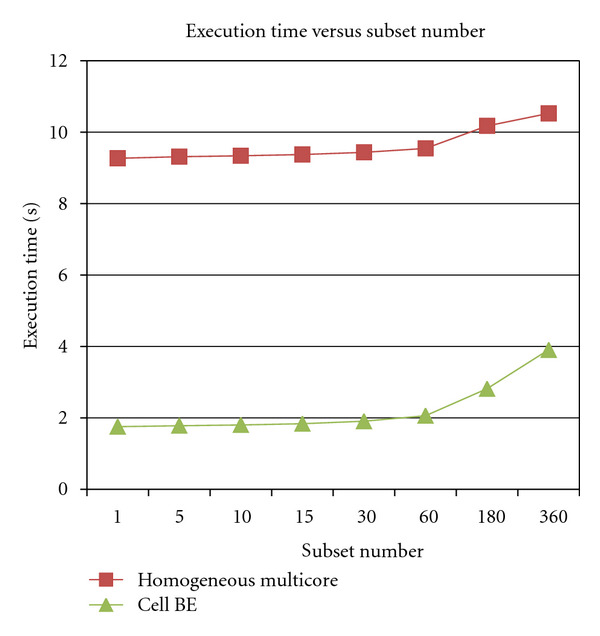
Computation time versus number of subsets for one iteration.

Figure 4 shows the sequential computation time with varying number of subsets for both the Cell processor (1 SPE) and the AMD Opteron dual-core processor (1 core). The figure shows that the number of ordered subsets impacts the processing time for both the Cell and the Opteron processor. In both cases, execution time increases with increasing subsets. This can be easily explained as follows. As the number of subset increases, the number of image update also increases. Since the image update is done by the PPE and has to be done sequentially, the sequential portion of the algorithm, therefore, limits the performance on the entire algorithm confirming Amdhal's law. As can be seen from the speed up curve, for one subset, the algorithm running on one SPE is over 5 times faster than on one core of the AMD Opteron processor. For 360 subsets, the Cell BE is 2.7 times faster than AMD Opteron processor. Note that for larger subsets, the number of DMA transfers between the local store and main memory increases on the Cell BE, increasing execution time. However, compared to AMD Opteron processor, the Cell BE still performs better.

**Figure 5:**
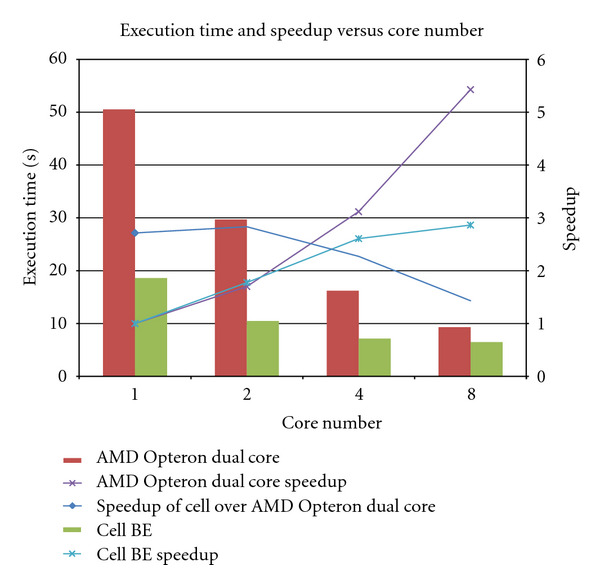
Computation time and speedup versus number of SPEs/cores for 20 subsets and 10 iterations.

Figure 5 shows the computation time and speedup for different number of SPEs and AMD cores. We set the number of subsets *T* = 20, the total number of projections, *Q* = 360, the total number of processors *P* = 8, to reconstruct the image for *I* = 10 iterations. Each subset is assigned 360/20 = 18 projection angles. Among the 360 projection angles, we can randomly select 18 angles for each of the subsets. However, in our algorithm, we follow the equation mentioned in Section 3. That is, the ordered subset OS_l_ is created by grouping the projections (PR_*q*_, 0 ≤ *q* < 360) whose indices *q* satisfy *q*mod *T* = *l*. Therefore, for the 360 projections, OS_0_ will consist of projections 0, 20,40,…, 340. OS_1_ will consist of projections 1,21, 41,…, 341. The algorithm starts with OS_0_. The 18 projection angles from OS_0_ are then subdivided and assigned to SPEs. Therefore, in Figure 5, for 8 SPEs, 360/(20∗8) projection angles are assigned to each SPE which performs forward projection, back projection, error correction, and rotation on their locally assigned data.

Since the Cell BE consists of 8 SPEs (processing elements or cores), our comparison on AMD Opteron is also for maximum of 8 cores. Figure 5 shows that the speedup on Cell BE is better than AMD Opteron processor when the number of processing elements used is less than 4. However, the speedup drops for Cell BE when more SPEs are used due to increased number of DMA transfers. This is due to the limited amount of local store available on each of the SPEs. As more SPEs are added, the number of DMA transfer increases since only a small amount of data can be DMAed in or DMAed out from main memory to local store and vice versa. This adds to memory latency and communication overhead. It was observed that the communication portion (including the DMA transfers and synchronization overhead) increased from 62% for one SPE to 86% for eight SPEs. The AMD HyperTransport technology attributes to the better speedup when more AMD cores are involved.

**Figure 6:**
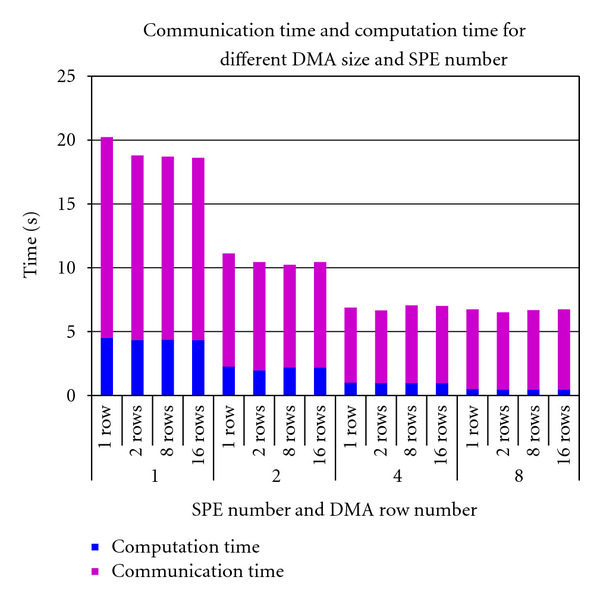
Computation time and communication time versus number of SPEs and number of image rows per DMA transfer for 20 subsets and 10 iterations.

Figure 6 shows the computation and communication times of the proposed algorithm for different DMA transfer sizes. We experimented with 1, 4, 8, or 16 image rows for each DMA transfer from main memory to the local stores and vice versa. As the figure indicates, the DMA transfers significantly add to communication cost dominating the total execution time of OS-SART on Cell BE. The communication/computation ratio is significant for larger SPEs. 

**Figure 7:**
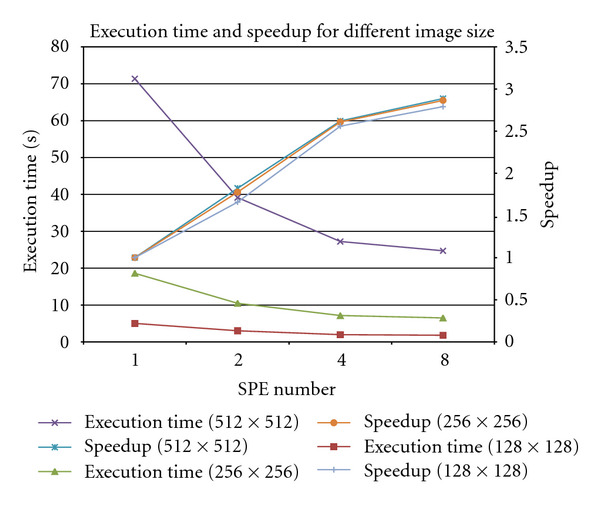
Computation time and speedup versus number of SPEs for different image sizes using 20 subsets and 10 iterations.

Figure 7 investigates the scalability of our algorithm for varying problem size and image size. As the number of SPE increases for a given problem size, the execution time decreases. The speedup of the algorithm for any image size on 8 SPEs is approximately 2.8, and the speedup increases as the number of SPE increases. Therefore, current implementation of the OS-SART with rotation-based algorithm is scalable with increasing problem and machine sizes. 

**Figure 8:**
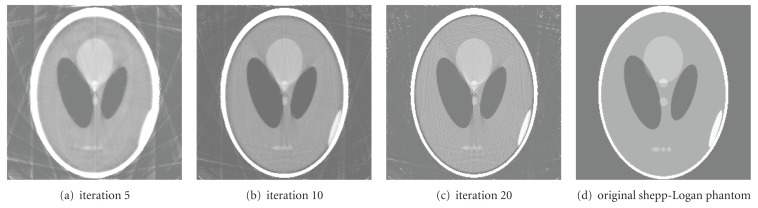
Reconstructed images at different iterations for 20 subsets.

Finally, Figure 8 illustrates the reconstructed images (256 × 256) obtained at different iterations. The number of subsets is 20. The image quality increases for more number of iterations. This result shows the accuracy of the algorithm. 

## 8. Discussion

High-performance computing is moving towards exascale computing. Heterogeneous parallel machines with accelerators such as graphical processing units (GPUs) have demonstrated their capabilities beyond graphics rendering or general purpose computing and are proved to be well suited for data intensive applications. However, the communication bottleneck for data transfer between the GPU and CPU has led to the design of the AMDs accelerated processing unit (APU), which combines CPU and GPU on a single chip. This new architecture poses new challenges. First, algorithms have to be redesigned to take advantage of this architecture. In addition, the programming models differ between vendors lacking the portability of algorithms across various heterogeneous platforms. With the future of general purpose computing moving towards APUs, it is important to understand the behaviour of these architectures on high performance computing applications.

As a stepping stone to understand the applications that can be studied on APUs, we have designed, developed, and implemented the OS-SART computed tomography algorithm on on-chip accelerator, the Cell BE. Cell BE has features similar to the APU. Therefore, we strongly believe that the algorithm design would remain intact without any modifications. That is the major impact of our algorithm design. Our algorithm carefully takes into consideration the different components of the Cell BE, the PPE (or CPU), and SPE (SIMD processors) and subdivides the tasks accordingly. Fine-grained data intensive tasks are offloaded to SPEs, while tedious tasks of data distribution and gathering are performed by the PPE. On an APU, the PPE tasks can be computed by the CPU and SPE tasks by the GPUs.

Porting of the algorithms from Cell BE to AMD APU is not straight forward due to the different programming paradigm. However, recently, OpenCL has been regarded as the standard programming model for heterogeneous platforms. The parallel code used in the paper can be rewritten in OpenCL providing easy portability onto the APUs.

One of the drawback of Cell BE is its limited memory storage on SPEs. The APU rectifies this with its large GPU memory size. The many cores available on the GPU will allow increased number of iterations for more accuracy for the same data size used in this paper without degrading the performance. We will also have the ability to experiment with larger data sizes.

Finally, in commercial CT machines, the Fourier back projection method is the algorithm of choice. This is partly due to the tremendous amount of computational power (required by iterative techniques) only obtained through supercomputers, making them unusable or unaffordable due to very high computational cost. However, with powerful general purpose computers in the market, it should be easy to develop iterative algorithms for use in real time to help medical practitioners with real time diagnosis.

## 9. Conclusions and Future Work

In this paper, we efficiently mapped the OS-SART algorithm using the architectural features of the Cell BE. One of the main drawback of the Cell BE is the limited memory storage on each of the SPEs. To circumvent this problem, we used rotation-based algorithm that incorporates a technique to calculate the projection angles using less memory. Though this was efficient, it also added to the number of transfers required to DMAin and DMAout the data from main memory to local store on SPE, which was a bottleneck as the number of SPEs increased. However, in comparison to a shared memory machine, the proposed algorithm on Cell BE performed much better.

The results showed that the number of ordered subsets impacts the sequential processing time on one SPE. However, Cell-based OS-SART on one SPE was five times faster than OS-SART on AMD Opteron core for one subset and one iteration. As the number of subsets increased with number of iterations, the speedup also increased. In the future, we will modify the algorithm using double buffering to overlap DMA transfers with computations in order to alleviate the impact of DMA transfers.

## Figures and Tables

**Algorithm 1:**
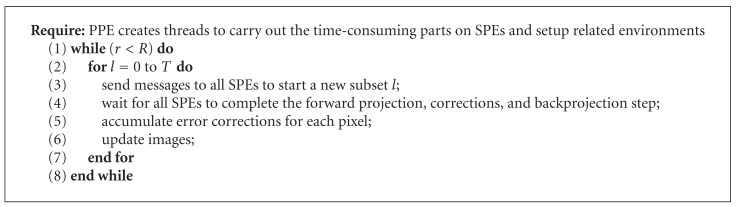
Parallel OS-SART on PPE.

**Algorithm 2:**
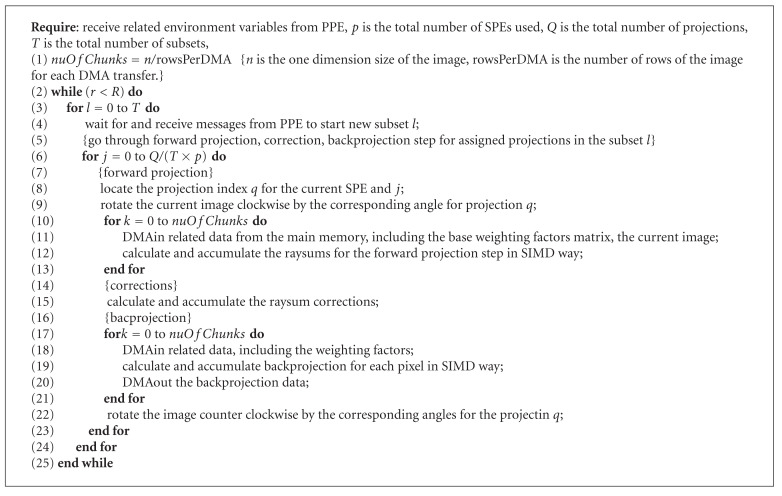
Parallel OS-SART on SPE.
